# Long noncoding RNA SNHG15: A promising target in human cancers

**DOI:** 10.3389/fonc.2023.1108564

**Published:** 2023-03-28

**Authors:** Niu Zhang, Tianyao Lei, Tianwei Xu, Xiaoteng Zou, Zhaoxia Wang

**Affiliations:** ^1^ Department of Oncology, The Second Affiliated Hospital of Nanjing Medical University, Nanjing, Jiangsu, China; ^2^ Department of Respiratory Medicine, Nanjing First Hospital, Nanjing Medical University, Nanjing, Jiangsu, China

**Keywords:** cancer, lncRNA, NSCLC, cancer resistance, target therapy

## Abstract

As oncogenes or tumor suppressor genes, lncRNAs played an important role in tumorigenesis and the progression of human cancers. The lncRNA SNHG15 has recently been revealed to be dysregulated in malignant tumors, suggesting the aberrant expression of which contributes to clinical features and regulates various oncogenic processes. We have selected extensive literature focused on SNHG15 from electronic databases, including studies relevant to its clinical significance and the critical events in cancer-related processes such as cell proliferation, apoptosis, autophagy, metastasis, and drug resistance. This review summarized the current understanding of SNHG15 in cancer, mainly focusing on the pathological features, known biological functions, and underlying molecular mechanisms. Furthermore, SNHG15 has been well-documented to be an effective diagnostic and prognostic marker for tumors, offering novel therapeutic interventions in specific subsets of cancer cells.

## 1 Introduction

Cancer was a major public health problem worldwide, with the burden of cancer continuing to rise (1–3). The 5-year survival rate and mortality rate of patients with advanced cancer were still poor. Therefore, it was imperative to explore potential regulatory mechanisms to identify novel biomarkers and develop effective therapeutic targets (4–6).

Cancer was an intricate multistep disease characterized by genomic instability that altered cellular homeostasis and promotes uncontrolled cell growth (7, 8). Several genomic mutations in cancer reside in regions that encode lncRNAs instead of proteins, which implicated lncRNAs in the onset and progression of cancer (9, 10).

LncRNAs were transcripts longer than 200 nucleotides in length that have no protein-coding potential, a cut-off that distinguished lncRNAs from smaller noncoding RNAs such as tRNA, miRNA, and Piwi-interacting RNAs (piRNA) (11–13). LncRNAs were considered to be important regulators of tissue physiology and disease processes including cancer (14). We considered them new cancer diagnostic and therapeutic gold mine in the future (15).

In recent years, the small nucleolar RNA host gene (SNHG) family of lncRNAs has garnered attention to play a role as oncogenes in various cancers (16–20). SNHG15, a member of the SNHG family of noncoding RNAs, had a length of over 860 kb in human chromosome 7p13 and was highly abundant and conserved among mammals (21). Based on existing findings about the relationship between the half-life of each mRNA and its physiological function, the studies raised the possibility that the stability of lncRNAs may reflect their underlying function. LncRNA SNHG15 was screened for a short half-life of non-coding transcripts that might be involved in cell proliferation (22). SNHG15 was initially proposed as a surrogate indicator of cellular stress due to its ability to sensitize human cells to cell death in response to various stresses (22, 23). As a novel molecule in the field of tumor biology, it was first reported to be highly expressed in gastric cancer and is well-known as a prognostic marker for liver cancer (24, 25). Moreover, it has been proven to be positively correlated with the malignant process and poor prognosis of renal cell carcinoma (RCC) (26), pancreatic cancer (PC) (27), bladder cancer (28), leukemia (29), etc. Human tumors have been evaluated concerning SNHG15 expression on a range of tumorigenic characteristics, including cell proliferation, apoptosis, invasion, and metastasis (30). In addition, abnormal expression of SNHG15 displayed a close correlation with tumor size, TNM stage, lymph node metastasis, and prognosis of tumor patients (31). Acquired drug resistance frequently led to solid tumor relapse and distant metastasis, and was considered to be the major reason for the failure of chemotherapy (32). Accordingly, understanding the molecular mechanisms of drug resistance was critical to allow the development of efficient therapeutic strategies with sustained anti-tumor effects (33). SNHG15 has been linked to chemoresistance such as gefitinib resistance in lung adenocarcinoma (34). As discussed above, SNHG15 seemed to play a crucial role in many types of malignancies. In this review, we summarized the recent progress made in the study of SNHG15 in tumors.

## 2 Expression of SNHG15 in cancer

The expression of SNHG15 was typically increased in multiple tumors, including lung carcinoma (LC), esophageal cancer, gastric cancer (GC), colorectal cancer (CRC), hepatocellular carcinoma (HCC), PC, kidney cancer, prostate cancer, breast carcinoma (BC), cervical and ovarian cancers (CC, OC), osteosarcoma (OS), oral cancer, glioma, and bladder cancer (**Table 1**). Interestingly, SNHG15 has an opposite expression in thyroid cancer, according to some researchers. Wu et al. stated that SNHG15 was highly expressed in papillary thyroid cancer (PTC) (66). However, in two papers published by Liu and his colleagues (67, 68), SNHG15 was reduced in thyroid cancer samples and acted as a tumor suppressor gene. The diversity of these studies could be partially related to tumor heterogeneity, different tumor origins, and cellular backgrounds, as well as limited numbers of specimens.

**Table 1:** Expression of SNHG15 in various cancers.

Cancer type	Expression in tissue	Sample size	Expression in cancer cells	Mentioned Cancer cell lines	Relative normal cell lines	Ref.
Lung cancer	Up	55	Up	H1703、H1799、 A549	BEAS-2B	(35)
	Up	24	Up	H358、H1299、H23、A549	HBEC3	(36)
	Up	49	Up	PC9、SPC-A1、A549、H1703、SK-MES-1	–	(37)
	Up	35	Up	A549、H460、SK-MES-1、Calu-3	NHBE	(38)
	–	–	Up	A549/GR、H1975/GR	–	(34)
Hepatocellular carcinoma	Up	58	Up	BEL-7402、HepG2、SMMC-7721、Hep3B	L-02	(39)
	Up	33	Up	HuH-1、HuH-7	L-O2	(40)
	Up	58	Up	SMMC‐7721、Hep3B、HepG2、Huh‐7	Lo2	(41)
	Up	152	Up	–	–	(25)
Colorectal cancer	Up	113	Up	HCT116、SW620、LoVo、SW480	–	(42)
	Up	108	–	–	–	(43)
	–	–	Up	SW1116、HCT116、SW480、SW620	–	(44)
	Up	36	Up	DLD1、HCT 116、HT-29、 LoVo、LS513、SW620、T84、RKO、SW480、Caco-2	HDFa	(45)
Gastric cancer	Up	30	Up	AGS、MNK-45、SNU-1	GES-1	(46)
	Up	106	Up	SGC7901、BGC823、MGC803、AGS、MKN45	GES-1	(24)
	Up	9	Up	HGC-27、MKN45	GES-1	(47)
Pancreatic cancer	Up	48	Up	AsPC-1、BxPC-3	HPDE6	(48)
	Up	60	Up	BxPC-3、PANC-1	HPDE6	(27)
Prostate cancer	–	–	Up	LNCaP、DU145、PC3	RWPE	(49)
Renal cell carcinoma	Up	62	–	–	–	(26)
	Up	96	Up	ACHN, OSRC-2, 786-O, 769-P and CAKI-1	HK-2	(50)
Breast cancer	Up	35	Up	BT-474、MDA-MB-468、SKBR-3、MCF-7	MCF-10A	(51)
	Up	42	Up	MCF-7/DDP、 MDA-MB-231/DDP	MCF-10A	(52)
	Up	58	Up	MCF-7、BT-20、ZR-75-1、MDA-MB-231	MCF-10A	(53)
	Up	30	Up	MDA-MB-231、MCF7、SK-BR3、T-47D	MCF-10A	(54)
Osteosarcoma	Up	–	Up	U2OS/DXR、MG63/DXR	HFOB	(55)
	Up	30	Up	MG63、U2OS、SaoS2、HOS	HFOB1.19	(56)
	Up	35	Up	143B、U2OS、HOS、MG63、 SaOS2	HFOB1.19	(57)
	–	–	Up	143B、U2OS	–	(58)
Oral squamous cell carcinoma	–	–	Up	SCC-15、SCC-9、SCC-25、HSC-2	NOK	(59)
Glioma	Up	40	Up	TMZ-R、TMZ-S	HMC3	(60)
	Up	–	–	glioma-induced hCMECs,	hCMECs in ACM	(61)
Ovarian cancer	Up	20	Up	CoC1、Angine、A2780、CAOV3、SKOV3、OVCA433	IOSE80	(62)
	Up	20	Up	SKOV3	IOSE80	(63)
Nasopharyngeal carcinoma	Up	50	Up	SUNE1、CNE1、CNE2、 HONE1	NP69	(64)
Cervical cancer	Up	28	Up	SiHa、HeLa、Caski、C-33A、MS751	HEKn	(65)
Bladder cancer	Up	30	Up	5637、UMUC3、J82、T24	SV-Huc-1	(28)
Thyroid cancer	–	–	Up	BHP5-16、BCPAP、K1、BHP2-7	Nthy-ori-3-1	(66)
	Down	50	Down	TPC1、FTC133、BCPAP、8505C	Nthy-ori-3-1	(67, 68)

–, not available.

## 3 Biological significance in the hallmarks of cancer

**Figure 1:**
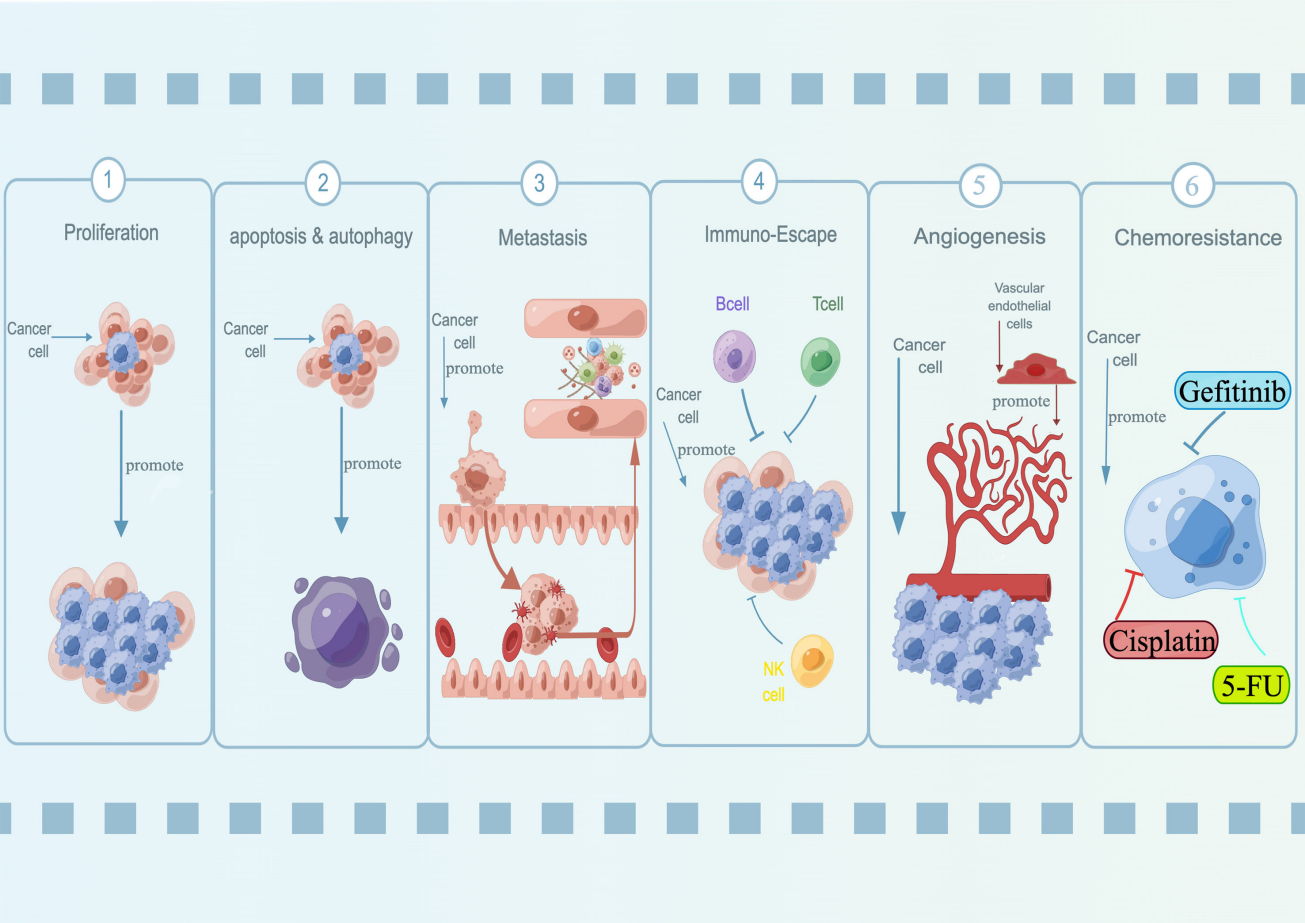
Oncogenic roles of lncRNA SNHG15 in a variety of diseases. SNHG15 has been identified for its fundamental role in regulating proliferation, apoptosis, autophagy, metastasis, immuno-escape, and chemoresistance.

During malignancy development, tumor cells acquired properties of stress-related conditions that enabled them to survive and adapted to the tumor microenvironment. These features were known as hallmarks of cancer, which were critical to their ability to form malignant tumors (69–71). Over the past few years, SNHG15 has been identified for its fundamental role in regulating oncogenes and tumor suppressors, as well as the underlying cancer characteristics (**Figure 1**; **Table 2**). Interestingly, not for common molecular sponge actions, Liu et al. found that miR-510-5p promoted thyroid cancer cell proliferation, migration, and invasion by suppressing SNHG15 (67, 68). Due to the fact that SNHG15 has only been reported as a tumor suppressor in thyroid cancer, the following functional studies concentrated on its involvement in cancer promotion.

**Table 2:** *In vitro* functional characterization of SNHG15 in cancer.

Cancer type	Effect on proliferation	Effect on apoptosis/autophagy	Effect on invasion/metastasis	Effect on immuno-escape	Effect on angiogenesis	Effect on chemore-sistance	Relative Cancer cell lines	Ref.
Lung cancer	promote	inhibit	promote	–	–	promote	A549/GR、H1975/GR、H358、H460、H1799、 A549	(34–38)
Hepatocellular carcinoma	promote	inhibit	promote	–	–	–	SMMC‐7721、HuH-1	(39–41)
Colorectal cancer	promote	inhibit	promote	–	–	promote	HCT 116、LoVo、SW620、SW480、	(42–45)
Gastric cancer	promote	inhibit	promote	promote	–	–	BGC823、MGC803、AGS、HGC-27、	(24, 46, 47)
Pancreatic cancer	promote	inhibit	promote	–	–	–	AsPC-1、BxPC-3、PANC-1	(27, 48)
Prostate cancer	promote	–	promote	–	–	–	LNCaP、PC3	(49)
Renal cell carcinoma	promote	promote	promote	–	–	–	ACHN、786-O	(50)
Breast cancer	promote	inhibit	promote	–	–	promote	SKBR-3、MCF-7、MCF-7/DDP、 MDA-MB-231/DDP、MCF-7、BT-20、MDA-MB-231	(51–54)
Osteosarcoma	promote	inhibit/promote	promote	–	–	promote	U2OS/DXR、MG63/DXR、SaoS2、HOS、U2OS、MG63、143B	(55–58)
Oral squamous cell carcinoma	promote	inhibit	promote	–	–	–	SCC-15、SCC-9	(59)
Glioma	promote	–	promote	–	promote	–	glioma-induced hCMECs,	(61)
Ovarian cancer	promote	inhibit	promote	–	–	–	SKOV3、OVCA433	(62, 63)
Nasopharyngeal carcinoma	promote	inhibit	–	–	–	–	SUNE1、CNE1	(64)
Cervical cancer	promote	inhibit	promote	–	–	promote	SiHa、HeLa	(65)
Bladder cancer	promote	–	promote	–	–	–	UMUC3、T24	(28)
Thyroid cancer	promote	inhibit	promote	–		–	BCPAP、K1	(66)
	inhibit	–	inhibit				TPC1、FTC133、8505C	(67, 68)

–, not available.

### 3.1 Proliferation

Tumors were typically formed and progressed due to unrestricted tumor cell proliferation resulting from either oncogene activation or tumor suppressor gene inactivation (72).

High expression of SNHG15 could up-regulate ZNF217 by adsorption of miR-211-3p, and inhibit miR-486 to promote CDK14 expression and ultimately stimulate the proliferation of NSCLC cells (36–38). *In vivo*, the xenograft model indicated that SNHG15 silencing dramatically inhibited NSCLC cell growth (38). In thyroid papillary carcinoma, exogenous overexpression of SNHG15 significantly blocked cell proliferation. SNHG15 served as a competitively endogenous RNA (ceRNA) in modulating the YAP1-Hippo pathway through binding with miR-200a-3p, among which YAP1 was a well-known oncogene (66). Moreover, SNHG15 has been well-studied in digestive system diseases. Chen et al. disclosed that SNHG15 manifested its oncogenic properties in the progression of GC by impairing miR-506-5p (46). A putative binding site for miR-506-5p was identified in the SNHG15 3’-untranslated region (3’-UTR) and further validated by the dual-luciferase reporter (DLR) assay. Through interaction with AIF, SNHG15, a bifunctional MYC-regulated lncRNA, induced the growth of LoVo and SW620 tumor cells in CRC (45). SNHG15 expression was significantly increased in liver cancer tissues and cell lines, whereas its downregulation inhibited *in vitro* tumor cell proliferation (39–41). By sponging miR-141-3p, SNHG15 could facilitate zinc finger E-box binding homeobox 2 (ZEB2) and E2F transcription factor 3 (E2F3) expression, thus mediating tumorigenesis (41). Several subsequent studies suggested that SNHG15 was also involved in the regulation of HCC cell proliferation through the miR-18b-5p/LMO4 axis and miR-490-3p/HDAC2 axis (39, 40).

The present results provided compelling evidence that SNHG15 functioned as an active regulator of cell cycle G1/S transition to promote cancer cell growth *in vitro* and *in vivo*. In pancreatic cancer, the knockdown of SNHG15 resulted in G0/G1 phase block, thereby inhibiting cell proliferation *in vitro*, similar to the roles of SNHG15 in ovarian cancer (48, 63). Mechanically, SNHG15 recruited EZH2 to suppress P15 and KLF2 expression and promoted pancreatic cancer proliferation (48). P15, one of the universal cyclin-dependent kinases (CDK) inhibitor proteins, has been revealed related to blocking cell cycle progression at the G0/G1 checkpoint (73). Molecular pathway analysis indicated a potential route for SNHG15 to promote ovarian cancer proliferation was by inhibiting tumor suppressor, miR-370-3p which leads to activation of CDK6 (63). CDK6 was a component of the core cell cycle complex that phosphorylates the corresponding proteins to drive cell proliferation (74).

Although the pathways or interactions by which SNHG15 promoted malignant cell proliferation are not consistent, it did appear to regulate cell proliferation through the function of ceRNA in numerous cancer types and its influence on cell cycle progression, which might represent a therapeutic vulnerability that warrants further investigation.

### 3.2 Apoptosis

Apoptosis, an evolutionarily conserved form of programmed cell death, played a massive role in the maintenance of tissue homeostasis by controlling cellular deletion to balance cell proliferation (75, 76).

Knockdown of SNHG15 significantly promoted the apoptosis of osteosarcoma cells, mainly due to a resulting increase in expression of apoptosis-related protein Bax, cleaved caspase-3, and downregulation of anti-apoptotic Bcl-2. Mechanistically, SNHG15 functioned as a ceRNA to sponge miR-346 and negatively regulate its expression in OS (56).

Suppression of SNHG15 increased the proportion of cells in the G0/G1 phase and apoptotic rate, which suggests that cell death eventually occurred through cell cycle arrest and subsequent apoptosis of cancer cells (38, 39, 51, 64). In breast cancer, SNHG15 induced caspase-dependent apoptosis *in vitro* and *in vivo* through the upregulation of Bax and Bcl-2 *via* the miR-411-5p/VASP axis (51). Silencing SNHG15 reduced the expression of CDK14 in A549 and H460 lung cancer cells, with CDK14 being the gene involved in regulating cell cycle progression (38, 77). Further in another study, SNHG15 was capable of upregulating the expression of the caspase-3 and PARP, elevating the ratio of Bax/Bcl-2, suggesting the involvement of intrinsic mitochondrial apoptotic pathway (37).

Based on functional analyses of multiple tumor types, SNHG15 interacted with multiple molecular mechanisms and signaling pathways to impair apoptosis. We anticipate that targeting apoptotic pathways that remain operative in specific types of cancer cells and restoring apoptotic defense mechanisms, which would result in substantial therapeutic benefits in the promising future.

### 3.3 Autophagy

In recent years, mounting studies have indicated that aberrant expression of lncRNA is related to cell autophagy in diverse cancers (78, 79). Autophagy was a catabolic process by which subcellular membranes undergo dynamic morphological changes that result in the degradation of cellular proteins and cytoplasmic organelles (80).

Notably, SNHG15-driven autophagy through negative regulation of miR-141, according to a study performed by Liu et al., appeared to contribute significantly to osteosarcoma development (57). Markedly, miR-141 repression alleviated the reduction of levels of Atg5, LC3-II, and the ratio of LC3-II/LC3-I and the rise in levels of p62 by SNHG15 knockdown in U2OS cells. Both ATG5 and LC3 were associated with autophagosome formation, while ubiquitin-binding protein P62 was an autophagy substrate, and degradation of P62 implies enhanced levels of autophagy (81, 82).

In addition to contributing to the origin of cancer, the ability to escape cell death also played a fundamental role in therapy resistance, relapse, and metastasis (83). Generally, SNHG15 facilitated the survival of malignant cells by promoting autophagy and was a promising target for anti-cancer treatments in patients against sensitive malignant cells.

### 3.4 Metastasis

Metastasis, a hallmark of cancer, was a complicated multistep process involving cell adhesion, invasion, and migration (84). It has been documented that SNHG15 modulates tumor metastasis primarily by regulating EMT, a pro-metastasis process that enabled cells to migrate more efficiently and invade the underlying mesenchyme (85).

Transcription of SNHG15 was regulated by MYC oncogene, and overexpression of SNHG15 promoted the invasion of colon cancer by interacting with AIF (45). In another separate research, SNHG15 maintained the stability of Slug in tumor cells by impeding its ubiquitination and degradation *via* interaction with the zinc finger domain of Slug (44). Since Slug was a key regulator of EMT, SNHG15 as a stabilizer of Slug was expected to serve an essential role in the regulation of EMT (44, 86). After the silencing of SNHG15, the expressions of E-cadherin and β-catenin were memorably increased, while the N-cadherin and Vimentin were decreased, suggesting that SNHG15 was also involved in the development of OSCC (59), bladder cancer (28), BC (54), and OC (63). Based on bioinformatics data, SNHG15 upregulation has been identified from tissue samples of CRC with liver metastasis, indicating that high expression of SNHG15 is significantly associated with liver metastasis of CRC (43).

As matrix metalloproteinases, MMPs have been intensively investigated in cancer invasion and metastasis due to their universal function of degrading the protein components in the extracellular matrix (ECM) (87, 88). Western blot assay exhibited that SNHG15 interference significantly decreased the expression of migration and invasion-related proteins VEGF, MMP-9, and MMP-14, further supporting the emphasis on SNHG15 in BC (51). For *in vitro* studies, SNHG15 knockdown resulted in reduced migration of GC (24, 46) and NSCLC (35, 37)cells. Subsequent experiments concerning that cells with SNHG15-depleted had lower expression of MMP2, and MMP9, indicating the role of MMPs in SNHG15-induced enhancement of migration and invasion (24, 37, 46). In addition to conventional cell-level analysis, tumor metastasis and invasion-related proteins were detected in nude mouse tumorigenesis tissues, and the results showed the expression of MMP2, MMP9, Sail1, and Vimentin was lower in tumor tissues derived from SNHG15-deficient BC cells, while the expression of E-cadherin was high. Kong et al. explored the molecular mechanism of SNHG15 in breast cancer, revealing that SNHG15 acted as a miR-211-3p sponge in carcinogenesis. In line with this, tail vein injection of stable SNHG15-depleted BC cells in nude mice resulted in reduced tumor cell colonization in the lung compared to the control group (53).

Transcription factors are closely involved in EMT, which is a fundamental phenotypic transition (89). In the nucleus, the NF-κB complex binds to sequence-specific gene promoters and modifies the expression of several target genes, including those encoding for transcriptional modulators involved in EMT (Slug, Snail, Twist), thus promoting migration and invasion (90). The expression of NF-κB related proteins detected by Western blot analysis showed that the expression levels of Snail1, Slug, and ZEB1 in RCC cells transfected with SNHG15 siRNA were decreased significantly compared with that in the control group (50). In general, studies of SNHG15 modulating NF-κB and NF-κB-mediated EMT in particular, have opened up avenues for the potential targeted oncogenic NF-κB signaling therapy in metastatic cancers.

### 3.5 Immuno-escape

Solid tumors have somehow managed to avoid the elimination of various weapons of the immune system or have been able to limit the amplification of immune lethality, thereby evading eradication (91). The various means by which cancer cells bypass the immune system include immune checkpoint inhibitions that accrue myriads of immunosuppressive molecules consisting of programmed death 1 (PD-1) or its ligand PD-L1 (92, 93).

Of note, SNHG15 was positively correlated with PD-L1, and overexpression of SNHG15 significantly reduced the apoptosis rate of HGC-27 cells after incubated with peripheral blood mononuclear cells (PBMC) for 24 h when compared to NC, suggesting that SNHG15 promoted the immune escape of gastric cells to PBMC by regulating the expression of PD-L1. Further analysis confirmed that upregulated SNHG15 inhibited the expression of miR-141, an inhibitor of PD-L1, leading to increased expression of PD-L1, which resulted in resistance of gastric cancer cells to immune response. This study suggested a novel lncRNA-mediated mechanism for gastric cancer cells to evade the immune response, and SNHG15/miR-141/PD-L1 has the potential to be a new target for gastric cancer therapy (47).

Cancer immunotherapy (CIT), primarily represented by PD-1/PD-L1 inhibitors, has become a fashionable treatment approach and made substantial breakthroughs in the treatment of multi-solid tumors (94–96). Under the control of tumor microenvironment complexity and plasticity, lncRNAs enabled tumor evasion from immune surveillance and uncontrolled development of metastasis and drug resistance (97). Targeting SNHG15 with specific inhibitors provided a strategy for studying and improving the efficacy of immunotherapy.

### 3.6 Inducing/accessing the vasculature

Tumor angiogenesis is a pivotal process in cancer progression, with glioblastoma generally considered to be one of the most vascularized tumors in humans (98). It has been shown that lncRNAs are involved in the regulation of tumor vascular endothelial cell function (99).

SNHG15 was highly expressed in glioma vascular endothelial cells and knockdown of the ncRNA inhibited proliferation, migration, and tube formation *in vitro*. Survival analysis approved that SNHG15 was a potential prognostic factor for patients with glioma and negatively affected the overall survival of patients with primary glioma (61). Tumor-derived expression of vascular endothelial growth factor (VEGF) was considered a leading candidate in tumor expansion and vascular function (100). Cdc42, a small GTPase that regulates cytoskeletal dynamics, cell shape, and many other cellular processes, was shown to improve VEGF-driven angiogenesis as well (101). Mechanistically, SNHG15 elevated VEGFA and Cdc42 expression by sponging miR-451 at the post‐transcriptional level, facilitating the angiogenesis of glioma. Based on this study, miR-451 silencing occurred *via* SNHG15, which has binding elements for miR-451 at its 3′‐UTR, leading to aberrant expression of VEGFA and Cdc42. Despite a significant decrease of luciferase activity in the wild-type VEGFA and Cdc42 upon overexpression of miR-51, there was no effect on the mutant type. In a nutshell, lncRNA SNHG15 promoted the growth of glioma microvascular endothelial cells primarily by positively regulating the miR-451/VEGFA/Cdc42 axis, thereby pointing to possible diagnostics and therapeutics based on the axis (61).

In light of the hypervascular nature of glioblastoma, it is promising that antiangiogenic treatment agents could be identified by exploring the regulatory factors of tumor-suppressor genes (102). As a whole, this finding reported by Ma et al. revealed a plausible mechanism responsible for tumor angiogenesis, a complex process with clear relevance to tumor progression and metastasis, thus supporting the notion that SNHG15 may be a novel target for clinical treatment for glioma patients.

### 3.7 Chemoresistance

Therapeutic resistance was a major challenge in cancer treatment; however, this could be improved by modulating key cellular signaling pathways that conferred drug resistance to increase the treatment sensitivity of tumors (103). Since SNHG15 was closely related to many cellular signaling processes, its expression could be regulated to improve tumor sensitivity.

Cisplatin was a commonly used drug in neoadjuvant chemotherapy and adjuvant chemotherapy for tumors, however, drug resistance posed a major challenge to the clinical application of cisplatin in cancer therapy (104, 105). SNHG15 was overexpressed in cisplatin (DDP) resistant breast cancer cells and tissues, enhancing DDP resistance of breast cancer cells by sponging miR-381 (52). In cervical cancer, both *in vitro* and *in vivo* studies have shown that SOX12-induced SNHG15 promotes cervical cancer tumorigenesis and resistance to cisplatin *via* the miR-4735-3p/HIF1a pathway (65). Likewise, SNHG15 suppressed cisplatin-induced apoptosis and ROS accumulation through the miR-335-3p/ZNF32 axis and was involved in p53-mediated cisplatin resistance in OS cells (58). Interestingly, SNHG15 might also target the miR-381-3p/GFRA1 axis to reduce apoptosis by inducing autophagy, leading to chemotherapy resistance to doxorubicin (55). Several findings above may provide new insights into the discovery of strategies for OS treatment. In studies investigating the resistance of glioblastoma to temozolomide (TMZ), overexpression of SNHG15 was associated with poor patient survival, and knockdown of SNHG15 effectively inhibited the tumorigenic properties of TMZ-R cells *via* the regulation of miR-627/CDK6 pathway, increasing TMZ-R cell sensitivity to TMZ as well as decreasing TMZ-R’s capacity to generate M2 GAM and glioma stem cells. In conclusion, the present study suggested that SNHG15 may serve as a prognostic marker for TMZ resistance, and Palbociclib, a CDK6 inhibitor, could be used as an adjuvant for overcoming TMZ resistance and shifting microglial cells towards an M1 polarization (60). In addition, knockdown of SNHG15 in gefitinib-resistant A549/GR and H1975/GR cells resulted in sponging miR-451 to up-regulate MDR-1 expression, thus modulating EGFR-TKI acquired resistance in lung adenocarcinoma (34). In studies targeting CRC, inhibition of SNHG15 sensitized LoVo and HCT116 cells to 5-FU, which was the basal chemotherapeutic agent for CRC treatment. Elevated levels of SNHG15 were correlated with the ability of CRC cells to cope with cytotoxic stress induced by 5-FU, which might be mediated in part by its interaction with AIF (45).

Overall, SNHG15 was up-regulated in drug-resistant cancer tissues and cell lines (34, 45, 52, 55, 58, 60, 65). Several findings above may provide new insights into the discovery of strategies for treatment. Overexpressed SNHG15 could promote the occurrence of drug resistance in tumors and reduce the sensitivity of cancer cells to chemotherapy drugs, suggesting its potential as a crucial prognostic factor to predict tumor drug resistance.

## 4 Clinical significance in cancer

Overexpression of SNHG15 suggested poor prognosis, which was dramatically related to larger tumor size, lymph node invasion, higher histologic grade, advanced TMN stage, inferior overall survival (OS), and disease-free survival (DFS). Multiple studies have declared that the up-regulation of SNHG15 has significant clinical significance and was expected to serve as a promising biomarker for early cancer diagnosis and prognosis. Besides, down-regulation of SNHG15 could inhibit tumor cell proliferation and metastasis, reduce drug resistance and increase apoptosis, indicating a potential molecular target for future tumor therapy.

### 4.1 SNHG15 serves as a diagnostic biomarker in cancer

The early and accurate diagnosis was undoubtedly crucial to prolong the lifespan of cancer patients, yet current methods do not fully meet the demand for early diagnosis (106, 107). Numerous studies revealed that serum lncRNA could serve as an effective biomarker for cancer detection (108, 109).

Han et al. collected serum specimens from patients with NSCLC, patients with benign pulmonary lesions, and healthy volunteers, respectively, and ROC analysis indicated that serum exosomal lncRNA SNHG15 might well distinguish all stage NSCLC, early-stage (I/II) patients or advanced stage (III/IV) patients from normal controls (110). Further, in combination with CEA, SNHG15 displayed higher accuracy in the early diagnosis of NSCLC. In pancreatic ductal adenocarcinoma (PDAC), the serum level of SNHG15 was significantly higher in patients than in healthy controls (111). With an AUC of 0.727, ROC curve analysis illustrated that serum SNHG15 level might serve as a biomarker for screening PDAC patients from controls.

Despite the considerable interest in the differential expression of SNHG15 for multiple cancers, SNHG15 was less likely to help distinguish between specific origins of tumors. These findings emphasized the need for SNHG15 in combination with other specific biomarkers that could further enhance the diagnostic value of different cancers.

### 4.2 SNHG15 serves as a prognostic marker in cancer

Despite the progression of innovative treatment technologies such as targeted therapy and immunotherapy, the prognosis of cancer remains dismal (112, 113). Profiling potential biomarkers for cancer prognosis and elucidating their functional roles and molecular mechanisms will greatly impact the precision treatment of patients.

It was shown that the aberrant expression of SNHG15 was closely associated with tumor prognosis (**Table 3**). High expression of SNHG15 in cancer tissue samples exerted an extremely promising potential to predict the adverse prognosis of cancer patients (25, 29, 43, 111, 114, 115). According to Zhang et al., SNHG15 expression was associated with histological grade, TNM stage, and venous invasion in HCC. An analysis of K-M data revealed that patients with high expression levels of LncRNA SNHG15 had poorer overall survival rates. In a brief, high expression of SNHG15 was an independent predictor of poor prognosis in patients with HCC (25). However, in thyroid cancer, the DFS time of patients with lower SNHG15 expression levels was significantly shorter than that of patients with strong levels (67). Moreover, several meta-analyses explored the potential associations between SNHG15 and prognostic attributes and clinicopathological parameters. Several literature findings revealed that SNHG15 overexpression increased the risk of short OS, DFS, and recurrence-free survival (RFS), but without significant heterogeneity (31, 116–118). Furthermore, SNHG15 expression was positively correlated with TNM stage, histological grade, lymphatic metastasis, and distant metastasis. Subgroup analysis confirmed that the high expression of SNHG15 was associated with a significant decrease in OS in patients with digestive cancer, but not LC (116). The study by Zhang et al. suggested that SNHG15 can be a particularly powerful molecular biomarker with prognostic potential in gliomas compared with other known related neoplastic diseases (31). Furthermore, whether there was an association between SNHG15 and tumor volume size remains controversial due to the different criteria for inclusion in the analysis.

**Table 3:** Involvement of SNHG15 in cancer prognosis.

Cancer type	Prognostic indicator	Associated clinical features	Ref.
Lung cancer	OS, DFS	tumor size, TNM stage, lymph node metastasis	(35, 37, 38)
Hepatocellular carcinoma	Five-year survival rate	TNM stage, lymph node metastasis, differentiation degree, vascular invasion, invasive depth	(39, 40)
Colorectal cancer	OS	tumor stage, tumor depth, lymph‐node metastasis, liver metastasis, TNM stage	(43–45)
Gastric cancer	OS, DFS	invasion depth, TNM stage, lymphatic metastasis, regional lymph node metastasis	(24)
Pancreatic cancer	–	tumor size, TNM stage, lymph node metastasis	(27, 48)
Renal cell carcinoma	OS, RFS	tumor stage, pathological stage, histological grade, metastasis	(26, 50)
Breast cancer	OS	tumor size, lymph node metastasis, pathological stage	(52, 53)
Glioma	OS	–	(60, 61)
Ovarian cancer	PFS	–	(63)
Nasopharyngeal carcinoma	OS	clinical stage	(64)
Bladder cancer	–	tumor size, tumor stage	(28)
Thyroid cancer	OS	gender, tumor size, TNM stage, lymph node metastasis	(66)
	DFS	age, pathology classification, clinical stage, tumor size, lymph node metastasis, distant metastasis	(67)

–, not available.

The upregulation of lncRNA SNHG15 was significantly associated with poorer prognosis and clinical features, suggesting that SNHG15 may be a novel prognostic factor in various cancers. However, the current deficiency was that most of the subjects included were from China, with small case numbers of certain cancer types and the sample size was still limited. Thus, subsequent prospective studies with high-quality and large-sample sizes were warranted to further confirm the prognostic role of SNHG15 in cancer.

### 4.3 SNHG15 serves as a promising target for cancer therapy

While decades of intensive research into cancer have been conducted, no effective strategy existed to drastically cut the rates of cancer recurrence and mortality (119–121). More interesting was that the SNHG15 expression appears to be differentially modulated in different cancer types and correlates with tumorigenesis, tumor aggressiveness, and stage of cancer, which makes it a candidate for cancer therapies.

The downregulation of SNHG15 and its associated miRNA network overexpression inhibited tumor progression, suggesting SNHG15 as a promising therapeutic target. SNHG15 mediated several events in CRC pathogenesis (42, 44). As well, overexpression of SNHG15 led to an increase in resistance of CRC cells through strong binding to the transcription factor MYC (45). Several *in vitro* studies indicated that SNHG15 silencing could significantly inhibit the proliferation, migration, and invasion of HCC and RCC cells, induce cell cycle arrest in G1/G0 phase and promote cell apoptosis (39, 122). Furthermore, suppression of SNHG15 inhibited breast xenograft tumor growth in the nude mouse model and reduced tumor weight, exhibiting a strong anti-tumor effect.

Despite these major advances, the oncogenic mechanisms of SNHG15 remain obscure. There was still a long way to go before its application in clinical treatment, including exploration and elucidation of the precise mechanism of action underlying the anti-tumor effect of SNHG15.

## 5 Regulatory networks of SNHG15

SNHG15 exerted oncogenic or tumor-suppressive effects in cancer through multiple molecular mechanisms. A better understanding of the regulatory network of SNHG15 in human tumors might provide neoteric insights regarding tumor pathogenesis and lncRNA-based diagnosis and treatment of human malignancies.

### 5.1 Upstream regulators of SNHG15

Accumulating evidence manifested that genetic alterations and key transcription factors contributed to lncRNA dysregulation in human cancers (123, 124). To our surprise, some upstream transcriptional regulators of SNHG15 have been preliminarily explored in recent years (**Table 4**).

**Table 4:** Upstream regulators of SNHG15 in cancers.

Cancer type	Associated upstream regulators	Relevance	Ref.
Lung cancer	ZEB1	positive	(34)
Colorectal cancer	MYC	positive	(45)
Cervical cancer	SOX12	positive	(65)
Multiple myeloma	m6A	positive	(125)
Renal cell carcinoma	cg00953154, cg03440944, and cg16459265	negative	(26)
Glioma	p53	negative	(126)
Osteosarcoma	p53	negative	(58)

Prior work declared that two resident E-box motifs are binding platforms for transcription factor MYC on the SNHG15. Chromatin immunoprecipitation (ChIP)-seq data from ENCODE confirmed that MYC is bound to these boxes in different cancerous cell lines. The depletion of MYC in the LoVo cell line resulted in a significant decrease in the level of SNHG15, stating that MYC can activate the transcription of SNHG15 in CRC cells (45). Notably, as predicted by the JASPAR database, ZEB1 could bind to the promoter region of SNHG15 to transcriptionally accelerate its expression in gefitinib-resistant lung adenocarcinoma (LUAD) cells (34). Silencing of the P53 gene in human osteosarcoma U2OS cells significantly increased SNHG15 expression compared to control (58). Consistently, fundamental experiments confirmed that the binding of P53 protein to the SNHG15 promoter was diminished in P53 knockdown cells. In ovarian cancer, tumor cells that were knocked down for SOX12 showed reductions in SNHG15 expression and vice versa. Luciferase reporter gene analysis corroborated the interaction between SOX12 and SNHG15 promoter. Taken together, SOX12 was involved in the transcription of SNHG15 overexpression (65). Ultimately in clear cell renal cell carcinoma (ccRCC), DNA hypomethylation might play a notable role in elevating SNHG15 transcription by substantially modulating its expression level. In the human medulloblastoma cell line DAOY, SNHG15 was modulated by EphrinA5-induced signal transduction, with EphrinA5 stimulation significantly reduced SNHG15 expression which might be relevant for the regulation of tumorigenic processes in the context of glioma (126).

The field of epitranscriptome analysis is becoming increasingly popular among scientists (127). It has been found that RNAs possess more than 170 types of chemical modifications required for their proper function in pre-mRNA splicing, nuclear exporting, transcript stability, and translation initiation (128, 129). N6-Methyladenosine (m6A), the most abundant internal chemical modification of eukaryotic mRNAs, was catalyzed by the m6A methyltransferase complex (MTC) and removed by FTO and ALKBH5 (130). The authors demonstrated that overexpression of lncRNA SNHG15 in myeloma cells rescued the cell proliferation inhibition, cell migration inhibition, and accelerated apoptosis caused by ALKBH5 knockdown (125). Based on renal samples from a cohort, the correlation between the SNHG15 expression and the methylation status of CpG sites was analyzed by gene microarray (26). Using the methylation data, the methylation levels of 3 sites (cg00953154, cg03440944, and cg16459265) were negatively associated with SNHG15 expression respectively, followed by validation in clinical samples.

Compared with the abundant research on downstream mechanisms, the upstream exploration of SNHG15 was still far from sufficient or complete. With the growing number of lncRNAs annotated in genomes by high-throughput sequencing technologies, the list of transcripts with unknown upstream transcriptional regulatory mechanisms was increasing, which provides the possibility for further exploration. Shortly, the cascades of transcriptional regulators upstream of SNHG15 were promising to be determined by fully mining the database and conducting co-expression network studies.

### 5.2 SNHG15 interacts with proteins

Several studies have highlighted that lncRNAs were able to regulate the expression of target genes by interacting with RNA-binding proteins (131, 132).

SNHG15 could function as protein decoys to recruit proteins in transcriptional gene regulation, either coordinately or in a complex. Ma et al. elucidated that SNHG15-mediated oncogenic effects are partly due to epigenetic inhibition of P15 and KLF2 expression (48). Experimental assays validated that SNHG15 recruited EZH2 to P15 and KLF2 promoters and inhibited transcriptions of these genes *via* modifying H3K27me3 in PC cells. In short, SNHG15 suppressed P15 and KLF2 expression to promote PC proliferation through EZH2-mediated H3K27me3.

In colorectal cancer, diverse experimental methods have confirmed that SNHG15 interacted with the Slug zinc finger domain and affected Slug protein levels in a post-transcriptional dependent manner (44). Certainly, these findings manifested a novel mechanism underlying the control of Slug stability, where SNHG15 interacted with and blocked Slug degradation along the ubiquitin-proteasome system, ultimately accelerating colon cancer progression.

AIF is a bifunctional protein that exhibits distinct subcellular localizations consistent with its known roles in cellular stress or apoptosis program (133, 134). After translation in the cytosol, AIF was transported to the mitochondria, where it has a strong impact on various cellular stress and survival pathways (135). Assays have confirmed that SNHG15 interacts with AIF based on protein translation, and then participates in the stress response of colorectal cancer cells to 5-FU. Given the full length of the AIF protein interacting with SNNH15, and the subcellular localization of the lncRNA, SNHG15 might interact with AIF in the cytosol, coupling it to the correct mitochondrial translocation and ultimately increasing the sensitivity of the cell to 5-FU (45).

AGO2 is an indicator protein of lncRNA acting as a sponge, which can form a ternary complex of ncRNA-miRNA-AGO2 protein with adsorbed miRNA (136). In hepatocellular carcinoma, RIP experiments confirmed that SNHG15 and miR-141-3p were preferentially enriched in AgO2-containing micronucleus proteins, while this suggests that SNHG15 and miR-141-3p interact in an AgO2-dependent manner (41). In a short, SNHG15 might serve as a scaffold for RNA binding proteins (RBP) to form RNA-protein complexes that ultimately affect protein translation and post-translational modifications.

Currently, available knowledge already reveals an intricate network of lncRNA-protein interactions whose deregulation is frequently associated with pathology. The interaction network was anticipated to expand, providing invaluable clues about lncRNA cellular mechanisms and their diseases-associated variations.

### 5.3 SNHG15 regulates various genes

In addition, there are some molecules reported to be mediated by SNHG15, including MMP2 and MMP9. Experimental results appeared that mRNA or protein expression of MMP2 and MMP9 were decreased when SNHG15 was blocked compared with the control groups respectively. In light of these data, MMP2/MMP9 were positively regulated by SNHG15 at both transcription and translation levels. In brief, ectopic expression of SNHG15 contributed to the proliferation and invasion of gastric cancer cells in part through the regulation of MMP2 and MMP9 (24).

### 5.4 SNHG15 serves as ceRNA

**Figure 2:**
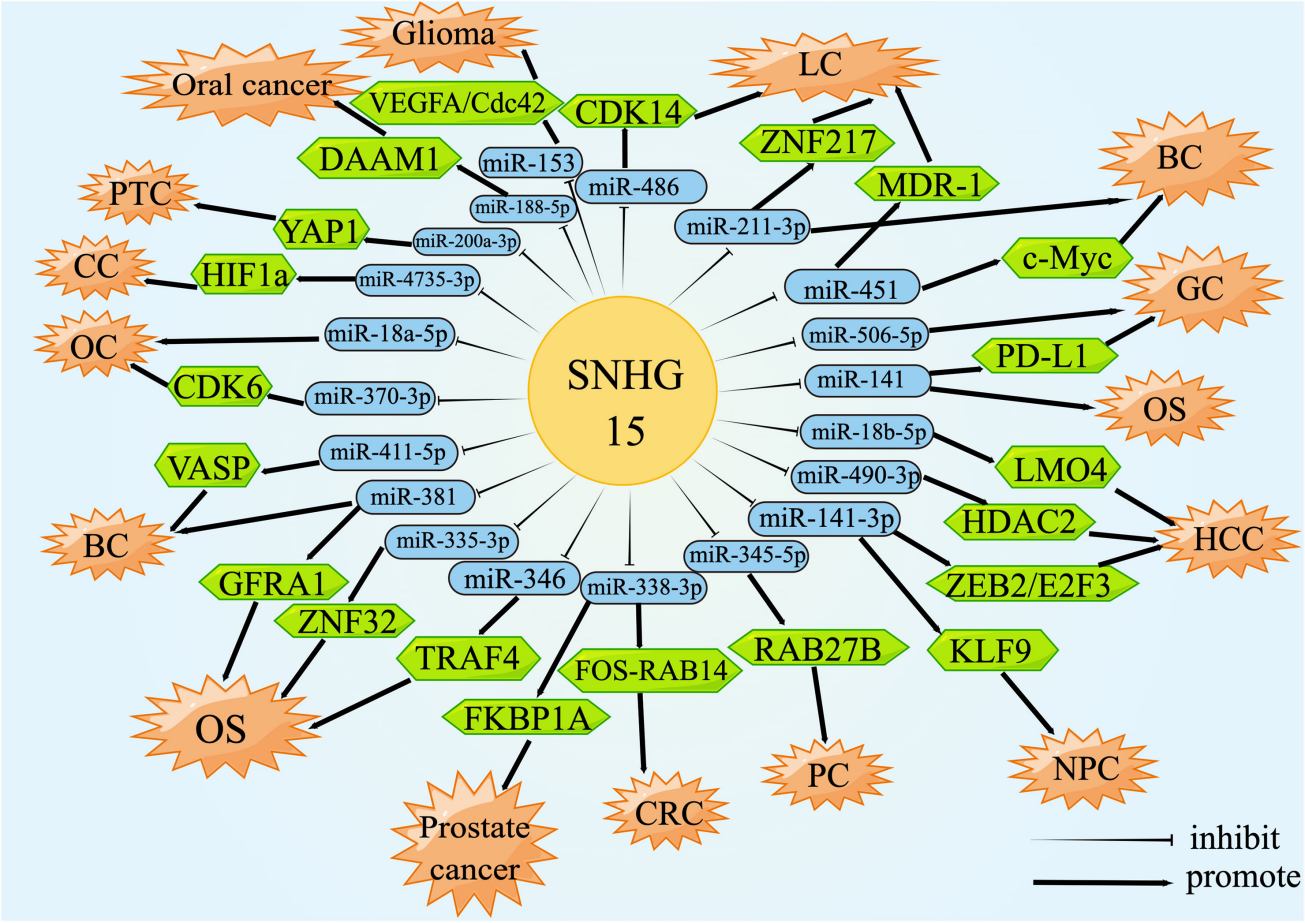
lncRNA SNHG15 function through ceRNA mechanism. SNHG15 could sponge miRNAs or serve as ceRNA by occupying the shared binding sequences of miRNAs, thus sequestering miRNAs and changing the expression of downstream target genes.

Theoretically, any type of RNA with miRNA response elements (MREs), including lncRNAs, could perform the function of ceRNA to degrade miRNAs and compete for the mRNA binding (137, 138). CeRNA was an important biological pathway that regulated RNA gene expression by interacting with the target of the mRNA 3’-UTR, resulting in adenylation, alterations in mRNA stability, and translation inhibition (139, 140). For instance, the upregulation of SNHG15 in prostate cancer tissues resulted in a rapid progression of the disease by increasing the expression of FKBP1A *via* miR-338-3p absorption. The luciferase activity of the wild-type SNHG15 reporter gene was appreciably reduced after transient co-transfection with miR-338-3p mimics but had no effect on the mutant. The miR-338-3p was capable of interacting directly with the non-coding region of FKBP1A, evoking a significant down-regulation of FKBP1A. Simply put, SNHG15 was involved in the regulation of the miR-338-3p/FKBP1A axis, thus encouraging the malignancy and tumorigenesis of prostate cancer (49). This review mainly summarized the lncRNA-miRNA-mRNA regulatory network formed by SNHG15 acting as a ceRNA in the whole process of sequential carcinogenesis (**Figure 2**).

### 5.5 SNHG15 regulates multiple signaling pathways

**Figure 3:**
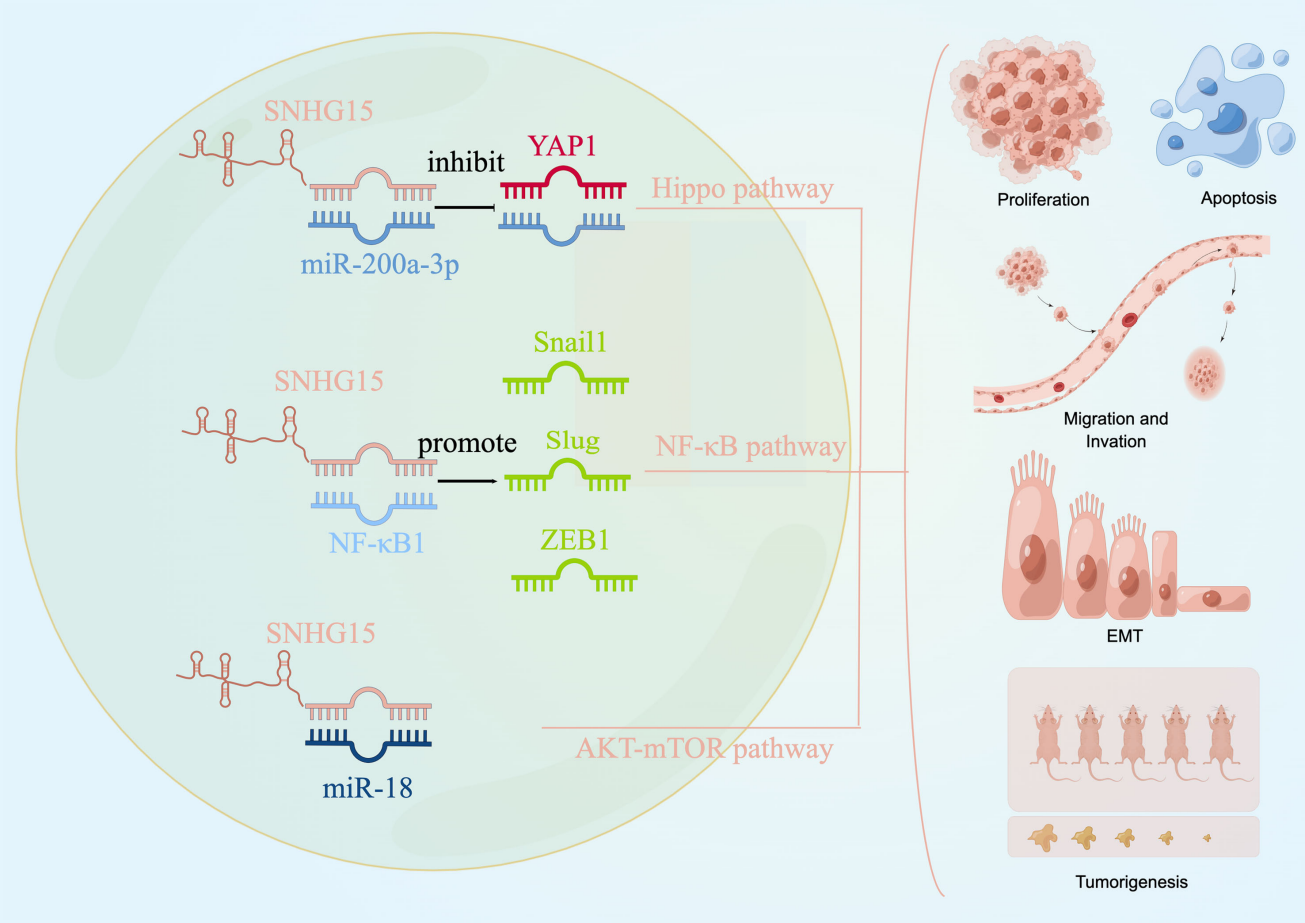
SNHG15 regulates multiple signaling pathways. SNHG15 exerted tumor-promoting effects by regulating YAP-Hippo and AKT-mTOR pathways.

We explored the literature on the signaling pathways involved in tumor development for SNHG15 to identify future serve as an early diagnostic biomarker or as a target for gene therapy or other curative treatments. Current studies from different groups suggested that SNHG15 exerted tumor-promoting effects by regulating YAP-Hippo, AKT-mTOR, and NF-κB signaling pathways (**Figure 3**).

### 5.5.1 YAP-Hippo pathway

The Hippo pathway is an evolutionarily conserved regulator of tissue growth and cell fate, and YAP1 is thought to be a major terminal effector of the pathway (141, 142). Not surprisingly, dysregulation of the Hippo pathway could induce tumors in model organisms and has been implicated in a broad range of human cancers. A recent study conducted by Wu et al. has confirmed the oncogenic regulatory axis of SNHG15/miR-200a-3P/YAP1 pathway in PTC progression (66). Wu and his colleagues pointed out that decreased SNHG15 expression effectively inhibited the briskness of the YAP1 signaling pathway and EMT process during PTC promotion. Moreover, MST1 and LATS1, the core factors of the Hippo pathway, are negatively regulated by SNHG15 at both the mRNA level and protein level in PTC cells. Given the frequent dysregulation of the Hippo pathway in cancer, targeting this way represented a very promising strategy for cancer treatment. The essential oncoprotein YAP, which was the ultimate common conduit of the Hippo pathway, was the most appealing therapeutic target. Considering the pro-tumor role of SNHG15/miR-200A-3p/YAP1/Hippo axis, combined targeting of the YAP1/Hippo signaling pathway might offer a promising treatment direction for PTC.

#### 5.5.2 AKT-mTOR pathway

The dysregulation of the PI3K/AKT/mTOR signaling pathway occurs most frequently in cancer patients and plays a critical role in driving tumor initiation and progression, as well as therapy response (143). Consistent with the changes of EMT markers *in vitro* study, the relative migrated and invaded cell numbers in the sh-SNHG15 group were significantly decreased compared to the NC group in ovarian cancer (62). Zhang et al. declared that SNHG15 participated in the biological process of ovarian cancer by directly targeting and inhibiting the miR-18a expression and thus regulating the AKT/mTOR signaling pathway. Western blot analysis on the protein levels of key molecules within signaling pathways showed that the expression of p-AKT and p-mTOR significantly increased in cells transfected with the SNHG15 vector. In a nutshell, this finding elucidated a plausible mechanism responsible for the constitutive activation of AKT-mTOR signaling during tumor promotion of OC, supporting the notion that SNHG15 might be a novel target for clinical treatment for cancer patients.

#### 5.5.3 NF-κB pathway

Since a eukaryotic transcription factor, NF-κB was involved in adaptive and innate immunity, inflammatory responses, cell proliferation, apoptosis, tumor growth, and differentiation (144). Aberrant activation of the NF-κB signaling cascade was recurrent in the initiation and progression of numerous human cancers, including kidney cancer (89). Based on the bioinformatics analysis, NF-κB might specifically bind to SNHG15, and Western blotting results substantiated that decreased SNHG15 expression effectively inhibited the active NF-κB signaling pathway and EMT processes in kidney carcinogenesis and led to a significant reduction in the protein expression levels of the EMT-related transcription factors Snail1, Slug, and ZEB1 (50). Previous studies have indicated that TNF-α, as an activator of NF-κB, was able to promote the intracellular membrane transduction process (90). Meanwhile, the migration and invasion ability of kidney cancer was enhanced by TNF-α stimulation, whereas the knockdown of SNHG15 attenuated this effect (50). These studies have identified that SNHG15 facilitated RCC proliferation, invasion, and migration through the NF-κB signaling pathway and by inducing the EMT process (50, 89, 90). An improved understanding of the molecular pathway implicated in the pathogenesis and progression of RCC has laid the foundation for the further diagnostic and therapeutic study of the disease.

## 6 Conclusion

Extensive evidence has highlighted that aberrant expression of human gene expression regulators such as lncRNAs plays a crucial role in tumorigenesis and progression (145, 146). In recent years, considerable progress has been made in elucidating the role of SNHG15 in various physiological and pathological processes. Nevertheless, SNHG15 was determined to be positively tied with unfavorable clinical parameters, resistance to therapy, and poor prognosis, revealing its potential as a promising biomarker for cancer diagnosis and treatment. For the relevant lncRNA SNHG15, pathway analysis of its immensely co-expressed protein-coding genes displayed a potential over-representation of cancer-related duties (50, 62, 66). Illumination of the molecular pathways and the interacting networks involved in SNHG15-mediated carcinogenesis will raise the understanding of its roles in the pathogenesis of multiple diseases and pave the way for in-depth knowledge of SNHG15, giving rise to clinical applications in the near future.

## 7 Future perspectives

Substantial empirical studies have demonstrated that tumor-derived exosome SNHG15 could carry multiple forms of tumor-associated information, which might be an untapped potential source of biomarkers for diagnostic or prognostic applications toward diverse cancers (25, 29, 111). In the future, the combination of SNHG15 and existing tumor markers is expected to greatly improve the accuracy of early diagnosis.

Cancer-related therapies targeting ncRNAs, including antisense oligonucleotides (ASOs), small interfering RNAs (siRNAs), short hairpin RNAs (shRNAs), and CRISPR-Cas9-based gene therapy have generated considerable interest in clinical management, with several above therapies showing promise in pre-clinical studies (147). The downregulation of SNHG15 inhibited tumor progression, suggesting SNHG15 could hold the promise of being a novel target for ncRNA-based pan-cancer therapies.

Since the conclusions are based on limited research so far, we still have a long way to go when it comes to understanding whether all tumors expressing high levels of SNHG15 are ideal candidates for diagnosis and treatment as a whole. Meanwhile, most of the findings documented here were derived from studies performed with established tissues and cancer cell lines. To fully explore the extent to which patients can benefit from SNHG15 monitoring and targeted treatment strategies, further stringent clinical validation was required before being deployed on patients with cancer.

## Author contributions

NZ and TL wrote the manuscript, TX and XZ prepared the figures and tables. ZW revised and designed the manuscript. All authors contributed to the article and approved the submitted version.
